# DMSO Reductase Family: Phylogenetics and Applications of Extremophiles

**DOI:** 10.3390/ijms20133349

**Published:** 2019-07-08

**Authors:** Jose María Miralles-Robledillo, Javier Torregrosa-Crespo, Rosa María Martínez-Espinosa, Carmen Pire

**Affiliations:** Departamento de Agroquímica y Bioquímica, División de Bioquímica y Biología Molecular, Facultad de Ciencias, Universidad de Alicante, Carretera San Vicente del Raspeig s/n-03690 San Vicente del Raspeig, Alicante, Spain

**Keywords:** dimethyl sulfoxide reductases, MoCo cofactor, redox reactions, biogeochemical cycles, *N*-cycle, phylogeny, nitrate reductase, perchlorate reductase

## Abstract

Dimethyl sulfoxide reductases (DMSO) are molybdoenzymes widespread in all domains of life. They catalyse not only redox reactions, but also hydroxylation/hydration and oxygen transfer processes. Although literature on DMSO is abundant, the biological significance of these enzymes in anaerobic respiration and the molecular mechanisms beyond the expression of genes coding for them are still scarce. In this review, a deep revision of the literature reported on DMSO as well as the use of bioinformatics tools and free software has been developed in order to highlight the relevance of DMSO reductases on anaerobic processes connected to different biogeochemical cycles. Special emphasis has been addressed to DMSO from extremophilic organisms and their role in nitrogen cycle. Besides, an updated overview of phylogeny of DMSOs as well as potential applications of some DMSO reductases on bioremediation approaches are also described.

## 1. Introduction

Molybdenum (Mo) is a transition metal that plays an essential role in metabolism in the three domains of life. It is a trace element; consequently, living beings require it in small doses. The biologically available form of Mo is the oxyanion molybdate (MoO_2_^−4^) from which organisms take up Mo along their daily life [1].

**Figure 1:**
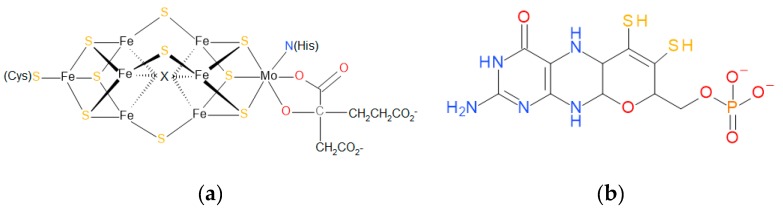
Molybdenum cofactors. (**a**) The iron-molybdenum cofactor (FeMoCo) of bacterial nitrogenases and (**b**) pyranopterin molecule from which the pterin-based cofactors (MoCo) are originated. Molecules drawn with BioVIA Draw 2019 [8].

Mo can be found as a cofactor at the active sites of enzymes in many organisms [2]. They are called molybdoenzymes and are involved in redox reactions of the global carbon, nitrogen, and sulfur cycles. Molybdoenzymes can show two different types of molybdenum cofactors: The iron-molybdenum cofactor (FeMoCo), which has only been identified in a bacterial nitrogenase, and the pterin-based cofactors (MoCo), which are widespread in all domains of life (Figure 1) [2,3,4]. This last cofactor is the one of interest in this review due to its relationship with dimethyl sulfoxide reductases (DMSO reductases). Another different cofactor with a molybdenum/copper heterometallic cluster has been identified in a protein called “orange protein” from *Desulfovibrio gigas*, but its function is still unknown at the time of writing this work [5,6]. Therefore, information about it and the hypothetical family it belongs to has not been included due to the lack of information.

The MoCo structure, as well as several genes participating in its biosynthesis, are conserved in plants, animals, fungi, archaea, and bacteria suggesting an evolutionary conserved pathway. Thus, at least the three first steps are shared by most organisms [1,7]: Synthesis of cPMP (cyclic pyranopterin monophosphate) from 5′-GTP (guanosine triphosphate), conversion of cPMP in MPT (molybdopterin, or also called metal-binding pterin, the metal-free form of the MoCo), and addition of molybdenum to form the MoCo (see Section 3 for more details). It is noteworthy that molybdenum is not active in cells until it forms the MoCo [4].

Apart from Mo, there are other transition metals with similar biological roles, tungsten (wolfram) (W) and vanadium (V) [9,10]. On the one hand, vanadium is present alternatively to the FeMoCo in some nitrogenases, as part of the iron-vanadium cofactor (FeVCo) [10]. On the other hand, tungsten is present as cofactor in tungsten enzymes, sharing a lot of resemblances with the MoCo of DMSO reductases [11].

Biochemically, Mo and W share several similarities, such as their analogous valence electron configuration in all oxidation states in almost all compounds and their similar atomic radii [12]. Whereas W does not have any role in eukaryotic organisms, Mo and W are relevant in prokaryotes [13]. Tungstate (WO_4_^−2^) is the biologically available form of W and is present in lower concentrations than MoO_2_^−4^ in oceans. Under euxinic conditions such as those characterizing hydrothermal vents (similar conditions to the ancient Earth), Mo forms MoS_2_, which is insoluble, whilst W forms soluble salts (WS_−4_^−2^) available for microorganisms [11,13,14]. This fact could explain why, under these conditions, we can find hyperthermophilic archaea, which are dependent on W instead of Mo [13]. Another remarkable aspect about the relationship between Mo and W (and directly related to their chemical similarity) is the fact that some enzymes, such as DMSO reductases, can use W instead of Mo in their cofactors when Mo is at lower concentration. It is probably due to the similarity between W cofactor (WCo, tungsten-enzymes) and Mo cofactor (MoCo, molybdoenzymes) [1,11,13,15]. However, some enzymes (even in the same family) can only use one or the other despite their similarities, so the discrimination mechanism in these enzymes would be very efficient [1,13].

This review is an attempt to compile the knowledge about the enzymes that contain Mo cofactors, focusing on the DMSO reductases family for their biological role in anaerobic respiration. The types of anaerobic respiration that they carry out, their position in the bacterial/archaeal phylogeny, the phylogenetic relationship between different DMSO reductases, and their role in the nitrogen cycle, as well as their potential applications in bioremediation, are reviewed.

## 2. Biosynthesis of the Bis-MGD Cofactor and Maturation of Molybdoenzymes

**Figure 2:**
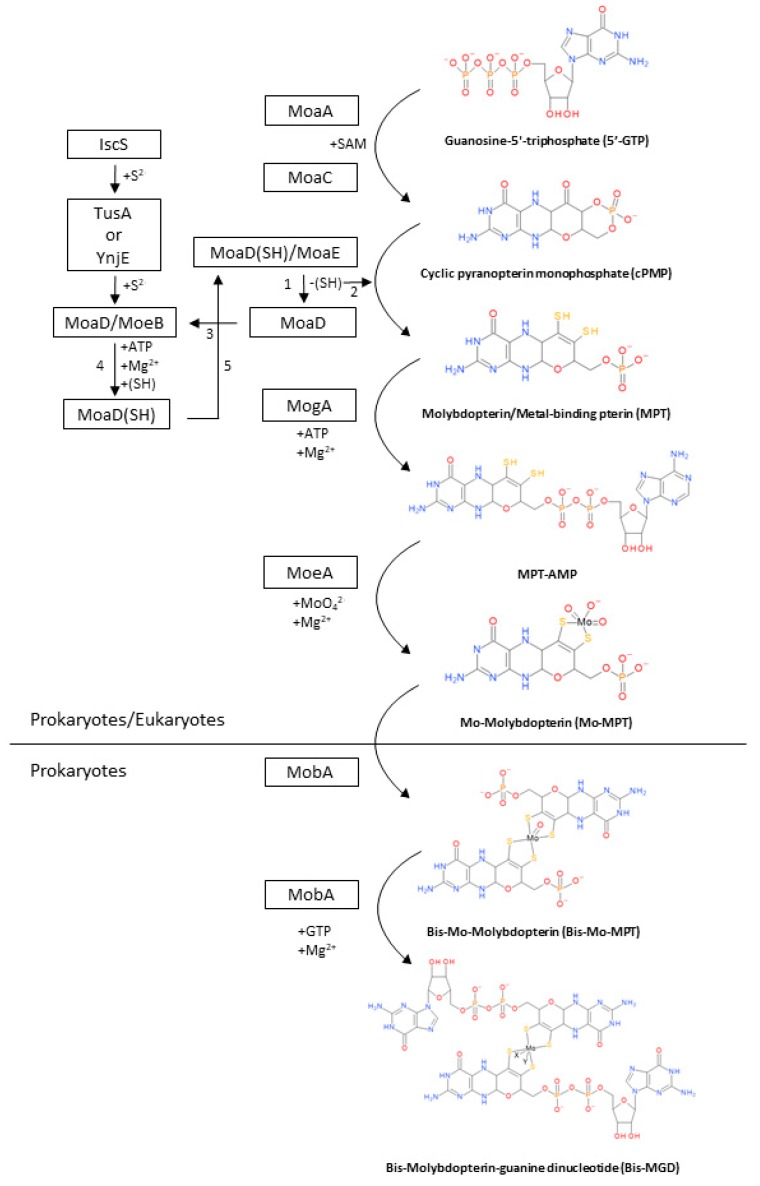
Biosynthesis of the bis-molybdopterin-guanine dinucleotide (Bis-MGD) cofactor. 1 and 2: Transference of the sulfur atom to the cPMP by active MPT synthase and dissociation of MoaD/MoaE. 3: Association of MoaD with MoeB. 4: Restoration of thiocarboxylate group and dissociation of MoaD/MoeB. 5: Reassociation of MoaD/MoaE (with the thiocarboxylate group). Molecules drawn with BioVIA Draw 2019.

The coordination and structure of the MoCo in DMSO reductases differentiate them from the rest of molybdoenzymes and tungsten-enzymes families and give to these enzymes their specific functionalities. However, despite the existence of a wide range of cofactors in molybdoenzymes and tungsto-enzymes, the first three reactions for the biosynthesis of the molybdenum cofactor are shared among all families [1,3,16,17]. DMSO reductases experience further modifications in the final steps after pyranopterin formation to achieve the formation of the bis-molybdopterin-guanine dinucleotide (Bis-MGD). This is the characteristic cofactor of that family in which the molybdenum atom is coordinated as we described in the section above [3]. A brief description of the biosynthesis process of the MoCo-DMSO reductases in prokaryotes is summarized in Figure 2. Molecular mechanisms described in this section are based on studies carried out on the model microorganism *Escherichia coli*. Almost all steps are conserved in the studied prokaryotes. However, this biosynthetic pathway may present slightly differences among organisms.

### 2.1. Synthesis of cPMP from 5′-GTP

Biosynthesis of molybdenum cofactor in all families initiates with the formation of the cyclic pyranopterin monophosphate (cPMP) from guanosine-5′-triphosphate (5′-GTP) in a process involving two enzymes: MoaA and MoaC (Figure 2) [3,17].

MoaA is a member of the radical *S*-adenosyl-l-methionine (SAM) superfamily and possess two [4Fe-4S] clusters (one in each monomer), which are oxygen sensitive [16]. MoaA is the first enzyme to act and it is responsible for forming the unstable and oxygen-sensitive intermediate 3′,8-cyclo-7,8-dihydro-guanosine 5′-triphosphate (3′,8-cH2GTP) (not shown in Figure 2) through a complex rearrangement of the 5′-GTP molecule [3,16,17,18,19]. MoaC acts in the conversion of 3′,8-cH2GTP to cPMP (the pyranopterin backbone). The cPMP molecule is free of sulfur but it already has the structure of three rings of MoCo [3].

### 2.2. Conversion of cPMP into MPT

The next step of the MoCo biosynthesis is the conversion of cPMP into molybdopterin/metal-binding pterin (MPT). This step is carried out mainly by the MPT synthase, but other accessory proteins are relevant too: MoeB, TusA, YnjE, and IscS. MPT synthase is an heterotetrametric enzyme constituted by two MoaD/MoaE heterodimers [3,17]. The MPT synthase catalyzes the reaction of incorporation of two sulfur atoms into the C1′ and C2′ positions of cPMP to generate diethylene groups (Figure 2). MoaD and MoaE have different activities besides forming the MPT synthase. MoaD have a thiocarboxylate group (-SH) at the C-terminus and is the sulfur donor, whereas MoaE have a binding pocket in which one molecule of cPMP can bind [20].

Once the MPT-synthase transferred the sulfur atom to the cPMP and convert it to the MPT, the MoaD/MoaE is inactive due to the loss of the thiocarboxylate group of MoaD [21]. This group must be restored to regain the MPT-synthase activity and this restoration is carried out by the accessory proteins. The restoration process starts with the separation of the MoaD without the thiocarboxylate group from MoaE and proceeds with the association of MoaD with MoeB (MoaD/MoeB) where the thiocarboxylate group is restored. Thus, when the thiocarboxylate group is restored, MoaD dissociates from MoeB and re-associates with MoaE restoring the activity of the MPT synthase (MoaD/MoaE) [21]. The sulfur that receives MoaD comes from the IscS sulfur transferase [21,22]. However, IscS do not directly interact with MoaD so, to transfer the sulfur atom to MoaD, it interacts with other proteins such as TusA or YnjE [23,24]

### 2.3. Metal Insertion in MPT to Synthetize the Mo-MPT

In the third step of the biosynthesis, molybdenum is inserted in the molybdopterin/metal-binding pterin molecule (MPT) as a molybdate ion (Figure 2). Two proteins are essential in this process to synthetize the MoCo: MogA and MoeA [3,17,25].

The actuation of these two proteins is sequential. Firstly, MogA activates the MPT for the molybdate insertion forming the adenylated intermediate MPT-AMP. Once the adenylated intermediate is formed, MoeA can insert the molybdenum atom and the binding of MPT-AMP is hydrolyzed, forming the Mo-molybdopterin (Mo-MPT). Mo-MPT is the last common intermediate of all families of molybdoenzymes and tungsten-enzyme.

### 2.4. Formation of Bis-MGD

The formation of the bis-molybdopterin-guanine dinucleotide (Bis-MGD) is the last step and it is exclusive of prokaryotes [3]. This step is carried out by two proteins whose genes (*mobA* and *mobB*) are cotranscripted: MobA and MobB. MobA is an essential GTP: Molybdopterin guanylyl transferase with a GTP binding site at the N-terminal and a putative Mo-MPT binding site at the C-Terminal. MobB is a non-essential protein whose functions are still unclear (X-ray studies suggest that it acts as an adapter in the formation of the Bis-MGD) [26,27,28,29].

The formation of Bis-MGD is a two-step process in which two molecules of molybdopterin guanine dinucleotide are ligated to a common molybdenum atom [30]. Firstly, MobA forms the bis-Mo-molybdopterin (Bis-Mo-MPT) intermediate from two Mo-MPT molecules [31]. These two molecules are connected through a molybdenum atom of one of them and the other molybdenum atom is released. The second and final step is the formation of Bis-MGD by the addition of GMP to each molecule of Mo-MPT (Figure 2) [29]. Finally, after the Bis-MGD formation, the cofactor must be inserted in the proteins by specific chaperones.

### 2.5. Maturation of DMSO Reductases

MoCo is a labile molecule easily oxidized. Due to its chemical nature, it is not common in nature as a free cofactor. Specific chaperones protect MoCo from oxidation in cell and sustain its insertion in the structure of the molybdoenzymes. The way in which chaperones identify and binds to their target molybdoenzymes depend on the type of protein: For exported molybdoenzymes, it binds to twin-arginine signal sequences (TAT system) and for cytoplasmic enzymes, the chaperones bind to other target sequences at the N-terminal different to TAT sequences [32,33,34].

Since the beginning of research on molybdoenzymes, the presence of chaperones in the coding operons from several molybdoenzymes has been highlighted, especially in the DMSO reductases family [26]. These chaperones tend to be specific from the protein encoded in the operon [34,35]. Nevertheless, in some DMSO reductases operons, the gene coding for a chaperone is absent [36]. In these cases, it has been suggested that the MobA protein can insert MoCo in the apo-proteins without modifications, or other chaperones from other molybdoenzymes can be shared for the maturation [34,37].

## 3. Families of Enzymes Containing Molybdenum or Tungsten

Nowadays there are more than sixty molybdoenzymes identified (bacteria and archaea) which are mainly divided into two groups according to their cofactors: MoCo and WCo (enzymes with WCo are not really molybdoenzymes but classically they are classified as such). Apart from them, there are FeMoCo-containing proteins including just one type of enzyme: The nitrogenases. They are present in diazotrophic organisms and have an essential role in the nitrogen cycle (catalysis of the reaction of biological dinitrogen (N_2_) fixation with the consequent reduction to ammonia (NH_4_^+^)) [10,38]. The cofactor of these enzymes contains MoFe_3_-S_3_X and Fe_4_-S_3_X cuboidal subunits, which share an X atom that can be H, C, or N [10].

Classically, molybdoenzymes with MoCo have been divided into three families based on its arrangement: Dimethyl sulfoxide (DMSO) reductases, xanthine oxidases, and sulfite oxidases [39]. As for the tungsten-enzymes, due to their similarity with DMSO reductases, some authors group them together. However, other more accurate classifications (based on the metal/cofactor structure), as stated by Maia and co-workers, classify the tungsten-enzymes in a separate family from DMSO reductases [11]. In this review, the classification presented is the following: Xanthine oxidases, sulfite oxidases, tungsten-enzymes, and DMSO reductases (Table 1).

In 2006, molybdoenzymes called mitochondrial amidoxime-reducing components (mARC) were described in the outer mitochondrial membrane in pig cells [40]. They are classified by some authors as members of a subfamily of sulfite oxidases. However, the lack of sequence similarity with enzymes of sulfite oxidase family as well as the biochemical and spectroscopic characterization of mARC, suggest that these enzymes (together with others: YcbX and YiiM) would form a new family [39,41,42]. This topic is now under discussion and is not treated in the review. The main features characterizing each family are described as follows, paying special attention to DMSO reductases.

### 3.1. Sulfite Oxidase Family

The family of sulfite oxidases is present in three domains of life and its main feature is the coordination of the MoCo by pyranopterin where Mo is covalently bound by a cysteine of the polypeptide chain of the enzyme (Table 1). They have also two oxo groups and the sulfurs of the cis-dithiolene group [11]. In the sulfite oxidases family, there are not only reductases, but also oxidases. Members of this family are enzymes involved in sulfite oxidation and others such as sulfite dehydrogenases and eukaryotic assimilatory nitrate reductases [43].

### 3.2. Xanthine Oxidase Family

Xanthine oxidases as well as sulfite oxidases are present in eukaryotes and prokaryotes. In this case, the MoCo have only one pyranopterin, which coordinates Mo through the sulfurs of the cis-dithiolene group, an oxo group, and a hydroxyl group, and depending on the enzyme, by a variety of groups: Sulfo, selene, oxo groups or S-Cu-S(Cys) (Table 1) [11,44,45]. In prokaryotes, the MoCo could be modified adding a CMP (cytidine monophosphate) group forming a molybdopterin cytosine dinucleotide. The best known and characterized enzymes of this family are xanthine oxidoreductases, aldehyde oxidases, and aldehyde oxidoreductases [11,44].

### 3.3. DMSO Reductase Family

In contrast to xanthine oxidases and sulfite oxidases, the DMSO reductase family is only found in bacteria and archaea and their presence in these organisms is really high [9]. More than 90% of Mo-utilizing organism possess enzymes of the DMSO reductase family. It suggests that is the most extensive family of molybdoenzymes in prokaryotic organisms [46].

DMSO reductases family cofactor is the bis-molybdopterin-guanine dinucleotide (Bis-MGD) and it is composed by two pyranopterin molecules (instead of one pyranopterin as in sulfite oxidases and xanthine oxidases families), which are conjugated with nucleosides: Cytosine or guanosine. In this family, the Mo atom in the MoCo is coordinated by four sulfur atoms of the pyranopterins rings and by an inorganic ion that could be selenium, oxygen, or sulfur atoms. In almost all cases, another ligand that has a role in coordination comes from an amino acid side chain that can be aspartate, serine, cysteine, and selenocysteine (Table 1) [9,11]. Depending on this amino acid, the DMSO reductases can be classified in three types: Cysteine or selenocysteine for type I, an aspartate for type II, and serine residue for type III [47].

Enzymes belonging to this family catalyze different types of reactions: Oxidation/reduction, hydroxylation/hydration, and oxygen transfer reactions [11]. Proteins such as assimilatory nitrate reductases, respiratory DMSO reductases, chlorate reductases, or formate dehydrogenase belong to this family.

### 3.4. Tungsten-Enzyme Family

The tungsten-enzyme family as well as DMSO reductases are only present in prokaryotic organisms. Both have the same coordination of the MoCo but replace the molybdopterins with tungstopterins (pyranopterins with W instead of Mo), probably due to the similarity between Mo and W (Table 1) [1,11,13,15,48]. This family has proteins with a wide range of functions. Some examples are acetylene hydratases, formylmethanofuran dehydrogenases, or formate dehydrogenases.

## 4. Connections between DMSO Reductases and *N*-Cycle

Among these enzymes, four of them are involved in anaerobic respiratory processes: Arsenate reductases, nitrate reductases, (per)chlorate reductases, and polysulfide reductases. Although respiratory nitrate reductases are the only enzymes directly connected to the nitrogen cycle (trough denitrification), the other three could be significantly involved in it too. This feature is due to the fact that some DMSO reductases are able to recognize more than one substrate under anaerobic conditions. Thus, some nitrate reductases can reduce not only nitrate, but also bromate, (per)chlorate, selenate, etc. [49,50]. Taking these observations into account, the main features characterizing nitrate, polysulfide, arsenate, and (per)chlorate reductions under anoxic conditions are described as follows.

### 4.1. Nitrate Reduction

Nitrate reduction to nitrite can occur under aerobic and anaerobic conditions as part of different reactions involved in the nitrogen cycle: (i) Assimilatory nitrate reduction (aerobic process), in which nitrate is used as nitrogen source for growth; (ii) dissimilatory nitrate reduction or nitrate detoxification (microaerobic or anaerobic processes), where nitrate is used as final electron acceptor to dissipate excess of reductant power; (iii) dissimilatory nitrate reduction to ammonium (DNRA) (microaerobic or anaerobic processes), in which the nitrite produced is further reduced to ammonium; and (iv) denitrification (anaerobic process), which uses nitrate is the final electron acceptor in a respiratory process in absence of oxygen.

**Figure 3:**
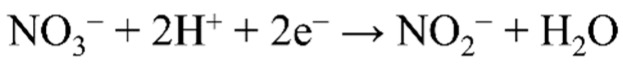
Reaction catalyzed by nitrate reductases. This is the first reaction in assimilatory nitrate reduction (catalyzed by Nas), Nitrate reductase (NR: eukNR, Nar, Nap, and Nas).

The enzymes catalyzing these reactions are generally called “nitrate reductases”. Based on their function, structure, and location, three different types of nitrate reductases are distinguished: Periplasmic nitrate reductase (Nap), respiratory nitrate reductase (Nar: membrane enzyme), and assimilatory nitrate reductase (Nas: cytoplasmic) [51,52,53]. Essentially, the three enzymes catalyze the same reaction (Figure 3) with different purposes: Nas catalyze the first reaction in the assimilatory nitrate reduction, Nap are usually involved in dissimilatory processes, and Nar catalyze the first reaction in denitrification.

As part of the processes discussed here, Nar and Nap proteins are the aim. These enzymes are structurally similar but have different locations: Periplasm and membrane, respectively. The Nap enzymes have been mainly described from mesophilic bacteria, and they are involved in different processes depending on the organism in which they are found [53]. On the one hand, Nap enzymes catalyze nitrate reduction to detoxify or to dissipate excess of reductant power. They have been described as heterodimers composed by a catalytic subunit (NapA) and a cytochrome c (NapB), which receive electrons from NapC, a membrane cytochrome c. Some authors describe the enzyme as a heterotrimer including NapC in the structure [51]. On the other hand, Nar enzymes are the key to reducing nitrate to nitrite under anoxic conditions. Not surprisingly, they are negatively regulated by oxygen, induced by the presence of nitrate, and unaffected by ammonium. In general, Nar enzymes are heterotrimers composed of a catalytic subunit (NarG or α subnunit) that binds a bismolybdopterin guanine dinucleotide (Bis-MGD) cofactor for nitrate reduction, an electron transfer subunit with four iron-sulphur centres (NarH or β subunit), as well as a di-b-haem integral membrane quinol dehydrogenase subunit (NarI or γ subunit). The NarG and NarH are membrane-extrinsic domains, whereas the NarI is a hydrophobic membrane protein, which anchors the NarGH complex to the membrane [51,54].

Nar enzymes may be the best studied nitrate reductases in extremophiles up until now. Several studies have described their isolation and characterization from halophilic or haloalkaliphilic archaea and thermophilic bacteria [55,56,57], whilst other studies were focused on the regulation of gene expression [55,58,59].

### 4.2. (Per)Chlorate Reduction

**Figure 4:**
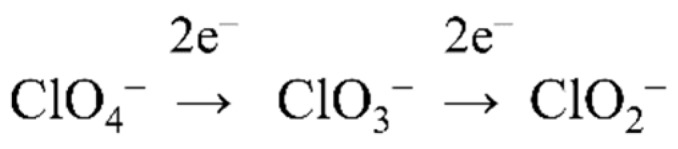
General scheme of perchlorate and chlorate reduction due to microbial activities. The first reaction is catalyzed by perchlorate reductases and the second one is catalyzed by chlorate reductases. Enzymes able to show both activities have also been described ((per)chlorate reductases).

Perchlorate can be reduced to chlorate by perchlorate reductases and then to chlorite by chlorate reductases. Perchlorate-respiring bacteria (PCRB) are ubiquitous in the environment, and are mainly facultative anaerobes and denitrifiers [60,61]. Perchlorate reductases isolated from PCRB react with both perchlorate and chlorate, and consequently, they are commonly named (per)chlorate reductases [62]. Chlorate reductases expressed by chlorate-respiring bacteria (CRB) do not reduce perchlorate [63]. All these enzymes are involved in the metabolism of oxochlorates as part of the biogeochemical cycle of chlorine. Thus, several microorganisms are able to carry out oxochlorates respiration under anoxic conditions following the scheme displayed in Figure 4 [64,65].

It has also been demonstrated that perchlorate and chlorate reductases isolated from some PCRB recognize nitrate as substrate [66], as well as several nitrate reductases, including those from extremophiles, are able to recognize perchorate and chlorate as substrates [50,67,68].

Structurally, perchlorate and chlorate reductases show several common features with respiratory nitrate reductases, i.e., they are heterotrimeric enzymes showing a large subunit (α) as a catalytic subunit, b subunit containing iron-sulfur clusters involved in electron transfer, and the smallest γ subunit (only found in chlorate reductases), which is homologous to the e heme β-containing subunit in selenate reductase, dimethyl sulfide dehydrogenase, ethylbenzene dehydrogenase, and archaeal p-type nitrate reductases.

Although (per)chlorate reduction was first described in mesophilic bacteria, recent studies have demonstrated that members of archaea domain as well as extremophilic bacteria are also able to carry out this pathway [69,70].

### 4.3. Arsenate Reduction

Arsenates are salts or esters of arsenic acid (H_3_AsO_4_), consisting of the ion AsO_4_^3−^. Whereas both arsenate and arsenite are strongly toxic to life, some prokaryotes use these compounds as electron acceptors or donors, respectively, for bioenergetic purposes via respiratory arsenate reductase, arsenite oxidase, and an alternative arsenite oxidase. Microbes dealing with these reactions usually inhabit contaminated environments and are commonly named “arsenate-reducing” bacteria and archaea [71,72,73]. Apparently, at least three families of arsenate reductase enzymes have arisen by convergent evolution [74]. They could be both periplasmic and membrane associated. Although the number of subunits and molecular masses differs, they all contain molybdenum and heterotrimeric forms predominate [75]. The expression of genes coding for arsenates reductases is usually switched on by parameters promoting detoxification mechanisms [72,76].

### 4.4. Polysulfide Reduction

At elemental sulfur reduction, the elemental sulfur (almost insoluble in water at ambient temperatures) may first turn into polysulfide ions, which theoretically could be reduced to hydrogen sulfide by sulfur-reducing bacteria. Thus, polysulfide (Sx (2-)) (soluble compound) could act as a possible intermediate directly used by bacteria in sulfur respiration. Sulfur reduction has been explored from a few sulfur reducers like *Clostridium* [77] or *Wolinella succinogenes* [78]. At the time of writing this review, the number of sulfur-reducing reductases purified and characterized is scarce and most of them have been isolated from hyperthermophilic bacteria or archaea [79].

It is interesting to note that all the enzymes described nowadays are heterotrimeric membrane-bound proteins showing similar pattern than those described for respiratory nitrate reductases and (per)chlorate reductases. For example, they are made of a large subunit (α) as a catalytic subunit, a β subunit containing iron-sulphur clusters involved in electron transfer, and a γ subunit (heme containing [80,81]. Polysulfide chemistry in natural environments is of high relevance due to a variety of relevant processes in which this compound is involved, including pyrite formation, organic matter sulfidization, isotope exchange among reduced sulfur species, and metal chelation [82].

## 5. Phylogenetics of DMSO Reductases: An Updated Overview

**Figure 5:**
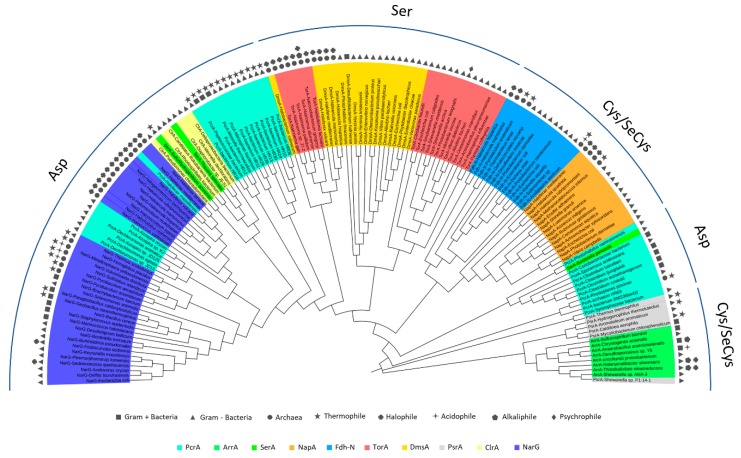
Phylogenetic tree built with 155 sequences of the catalytic subunit from 10 respiratory-related enzymes belonging to the dimethyl sulfoxide reductases (DMSO) reductase family, together with a brief description (legend) of the organism from which each sequence belongs. Phylogenetic tree was built with Clustal Omega and iTol v4 software.

In order to explore phylogenetic relationships between DMSO reductases, especially in extremophiles, a total of 155 available sequences coding for the catalytic subunit of respiratory-related DMSO reductases (including bacteria and archaea) have been selected and analyzed. Figure 5 displays the phylogenetic tree including enzymes belonging to the three types of DMSO reductases. Alignments have been done using Clustal Omega [83]. This software is based on the HHalgorithm described by Söding [84]. The alignments have been used to build a phylogenetic tree using the neighbour joining method from Clustal Omega. Manipulation and annotation of the phylogenetic tree has been done with the online tool “Interactive Tree Of Life (iTol) v4” [85]. Protein sequences were acquired from the protein database of NCBI.

Polysulfide reductases (PsrA), respiratory arsenate reductases (ArrA), periplasmic nitrate reductases (NapA), and formate dehydrogenases N (Fdh-N) represent the type I in the tree. Type II is represented by respiratory nitrate reductases (NarG), perchlorate reductases (PcrA), respiratory selenate reductases (SerA) and chlorate reductases (ClrA). Finally, type III includes DMSO reductases (DmsA) and trimethylamine N-oxide (TMAO) reductases (TorA). All enzymes are grouped mainly by this characteristic but type I enzymes PsrA and ArrA form a monophyletic group different from type I NapA and Fdh-N, while PcrA enzymes are also divided in different clades.

Polysulfide and respiratory arsenate reductases appear related in the tree, belonging to the DMSO type I. Their analogy has been reported in other studies, concluding that their catalytic subunits are similar [86,87]. It is remarkable the fact that members of the DMSO reductases family can use (with less effectivity) other alternative substrates, which are specific from other representatives. However, no dual activity between PsrA and ArrA has been reported despite their similarity. It has been proposed that arsenate reductase evolved from a Psr ancestor and the evolution modified an enzyme of the sulfur respiration pathway, the ancestor of the Psr, to create the arsenate reductase [86].

Periplasmic nitrate reductases and formate dehydrogenases N [86,88] form a monophyletic group. This relationship has already been reported and it is not only due to being part of the same type but also because of the presence of a highly conserved lysine residue. It has a role in the electron flow in redox reactions in both enzymes [89,90]. In NapA and Fdh-N clade, an early divergence between extremophilic archaeal and mesophilic bacterial NapA and Fdh-N is observed but no comparative research about it has been reported yet.

**Figure 6:**
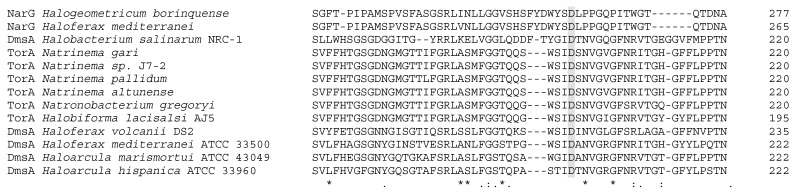
Clustal Omega alignment of halophilic respiratory nitrate reductases (NarG), TMAO reductases (TorA), and DMSO reductases (DmsA). In grey, aspartate which coordinates Mo atom in haloarchaeal NarG and DmsA aligned with possible coordination aspartate from haloarchaeal TorA.

Type III enzymes are grouped in two clades constituted by DMSO reductases and TMAO reductases from bacteria and archaea. Dual activity has been described in DMSO reductases, as in the case of the *Escherichia coli* DMSO reductase that can reduce TMAO and other N-oxides [91,92]. In contrast, no DMSO reductase activity has been found in biochemically characterized TMAO reductases [92]. The most remarkable aspect is the early divergence of DMSO reductases and TMAO reductases from halophilic archaea, which are completely separated from the mesophilic bacterial enzymes. Studies in the halophilic archaea *Halobacterium salinarum* proposed that the Mo amino acid ligand in the MoCo of DmsA is an aspartate residue in all halophilic archaea instead of a serine residue as in mesophilic bacteria [35,93]. Alignments presented here also suggest that halophilic TorA could have an aspartate residue coordinating the Mo atom. Figure 6 shows the alignment of halophilic two NarG with all the haloarchaeal DmsA and TorA in which the Mo-coordination aspartate from NarG and DmsA is aligned with TorA. This could explain why haloarchaeal TorA and DmsA are in a different clade from mesophilic TorA and DmsA. Taking this into account, haloarchaeal enzymes TorA and DmsA should be classified as type II DMSO reductases due to their MoCo coordination. Despite the above, experimental procedures are needed to confirm the functional implications of this feature.

Type II DMSO reductases include enzymes with great versatility in the use of different non-specific substrates [50,62]. Respiratory selenate reductases together with chlorate reductases constitute a clade, which indicates a great homology between both enzymes. This relationship is not well studied but it is reported that respiratory selenate reductase from the betaproteobacteria *Thauera selenatis* also have chlorate reductase activity [94]. The distance between the perchlorate and chlorate reductases indicates that they are different enzymes with less degree of homology, showing different specificity, since the perchlorate reductase can reduce perchlorate and chlorate, while the chlorate reductase can reduce only the latter [95,96].

NarG and PcrA clade is close to respiratory selenate and chlorate reductases. Archaeal and bacterial NarG enzymes are separated into two groups. Archaeal nitrate reductases differ from bacterial by the presence of the TAT exportation signal at the N-terminal. Due to that signal, archaeal NarG is facing to periplasm, whereas bacterial NarGs are facing cytoplasm [49,97]. Nevertheless, what is more surprising is that haloarchaeal NarG is closer to perchlorate reductases of a specific order of betaproteobacteria (*Rhodocyclales*) than the thermophilic archaeal and bacterial NarGs. This relationship has not been discussed yet, maybe because in other phylogenetic studies, PcrA sequences are scarce or are not present [86,88,98,99]. In the phylogenetic tree displayed in Figure 5, 29 PcrA sequences were analyzed. Furthermore, apart from the perchlorate reductase clade related to NarG, there are two more separated clades of perchlorate reductases: The closer clade constituted only by thermophilic perchlorate reductases and other distant clade involving mesophilic perchlorate reductases.

It has been reported that the ability to utilize perchlorate in perchlorate-reducing bacteria is conferred by a horizontally transferred piece of DNA: The perchlorate reduction genomic island. In a study of 13 genomes of perchlorate-reducing bacteria from four different classes of Proteobacteria, the authors described how the island varies considerably in genetic content, among the different phylogenetic classes, what gives rise to the phylogenetic classification of these enzymes [99].

**Figure 7:**
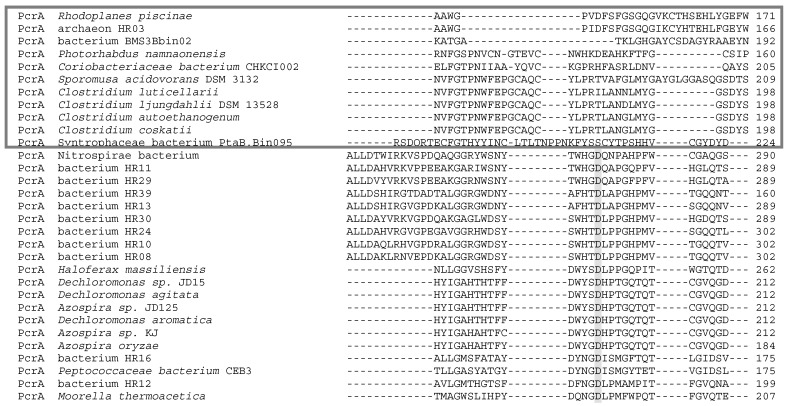
Clustal Omega alignment of all PcrA proteins. Aspartate, which coordinates Mo atom, is in grey. Grey box represents the separate clade of PcrA in which their aspartate is not aligned with the aspartate from the two other groups of PcrA.

The genomic context of the selected enzymes in this work has not be analyzed; consequently, the content of the genomic island is unknown. Nevertheless, sequence alignments of the PcrA of three groups have shown that in the region of conserved Asp residue for Mo coordination, the alignment differs in each group. The sequences of polypeptides annotated has PcrA but phylogenetically closest to type I DMSO reductases are very different to the other two groups, and indeed the Asp residue is not clearly identified in the alignments (Figure 7). So, more research will be necessary to clarify the classification of this group of enzymes.

The sequences of thermophilic PcrA also differ from the other two groups, some of them have longer chains (up to 1166 amino acids residues) and the aspartate ligated to Mo is clearly identified in the alignments. This group of PcrA shows an ancestral origin with respect the other type III DMSO reductases. Given that perchlorate probably existed on Earth in early geological times, it is possible to conclude that the capacity of thermophilic bacteria to respire perchlorate may have developed very early. The third cluster of PcrA corresponds to the *Rhodocyclales* betaproteobacteria. These chains form a monophyletic clusterwith NarG, as has been previously described [95]. NarG from haloarchaea are closer to this group of PcrA than to bacterial NarG. The reason for this could be because PcrA and haloarchaeal NarG are periplasmic subunits and therefore both contains a TAT signal that is absent in the NarG from bacteria (facing the cytoplasmatic side). Sequence homology is greater between haloarchaea NarG and bacterial PcrA than with bacterial NarG, and their structures will also probably be. This close relationship had been previously described with concatenated PcrAB sequences that were aligned to three of Archaeal periplasmic nitrate reductases [99].

PcrA and NarG can catalyze the reduction of different electron acceptors as bromate, chlorate, perchlorate, nitrate, and iodate. The bacterial enzymes have been described as related enzymes that have evolved from a common ancestor and diverged to adjust their activities to different environments. A perchlorate reductase would have evolved to have high affinity for perchlorate, whereas a nitrate reductase would have evolved to maintain low affinity for perchlorate [100]. When the structures of Escherichia coli NarG and *Azospira oryzae* (*Azospira suillum*) PcrA were compared, some differences at the substrate access hydrophobic tunnel were identified. Thus, in position 461, there is an aromatic Trp residue in PcrA, whereas in NarG, there is a Glu residue. It is postulated that this difference could explain the substrate preference of each enzyme [100]. The respiratory NarGH from the haloarchaeon *Haloferax mediterranei* has been studied and its chlorate and perchlorate activity were determined. In the genome of this haloarchaeon, no perchlorate or chlorate reduction system has been detected. *H. mediterranei* is then unable to grow in anaerobic medium with (per)chlorate as unique electrons acceptors. However, NarGH can catalyze the reduction of chlorate and perchlorate with an efficiency comparable to nitrate reduction, even though it shows a higher specificity towards nitrate than chlorate (Km values of 0.8 and 2.4 mM, respectively). Besides, cells previously grown anaerobically in the presence of nitrate were able to use chlorate as final electron acceptor [49].

To analyze the putative binding substrate site, a structural model of the *H. mediterranei* respiratory nitrate reductase has been constructed. The amino terminal sequence of NarG subunit (NCBI Protein database ID: WP 004056332.1) was analyzed to detect the TAT signal, and the first 66 amino acids were removed before modelling the structure. The 3D structure model was generated by I-TASSER software [101,102]. Its algorithm consists of three consecutive steps of threading, fragment assembly, and iteration. The server generates five models and, with a C- score of 0.76, the best predicted structure model of NarG from *H. mediterranei* was selected. The overall model predictions were evaluated by using TM-score and RMSD, whose estimated values were 0.82 ± 0.09 and 7.0 ± 4.1 Å, respectively. I-TASSER results show that the protein structurally closer to NarG from *H. mediterranei* was PcrA from *A. oryzae*.

**Figure 8:**
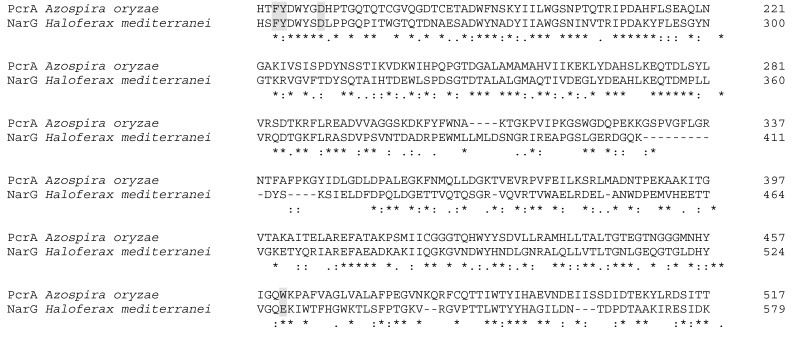
EMBOSS Needle alignment of PcrA (*A. aryzae)* with NarG (*H. mediterranei*). In grey are the residues of the hydrophobic tunnel of each enzyme. Trp461 from PcrA changes to Glutamate in NarG.

**Figure 9:**
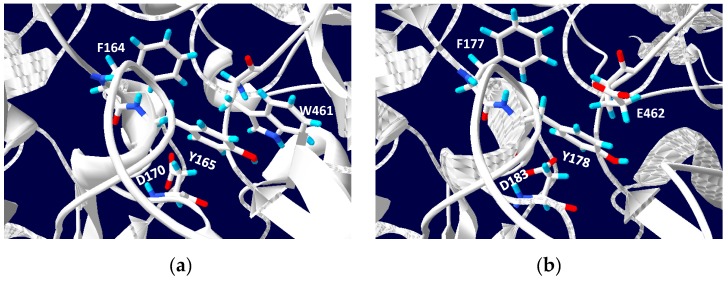
(**a**) Structure of the closed tunnel in PcrA from *A. oryzae* (PDB ID:4YDD). The gate residues F164, Y165, and W461 are shown in stick. (**b**) Model of NarG from *H. mediterranei*. The same positions of the hydrophobic tunnel in PcrA are showing in NarG structural model. W461 is substituted by E462 in the halophilic NarG. Blue: Nitrogen; Cyan; hydrogen; Red: oxygen.

Alignment between NarG from *H. mediterranei* and PcrA from *A. oryzae* shows that both enzymes are very similar, with 44% identity (Figure 8). Inspection on residues that could constitute the substrate access hydrophobic tunnel in *H. mediterranei* NarG model displayed that, as in *E. coli* NarG, the enzyme from *H. mediterranei* has a Glu residue instead a Trp, which would determine its preference for nitrate (Figure 8 and Figure 9).

The inability of several denitrifiers to grow using (per)chlorate could be due to the failure to induce nitrate reductase in the presence of chlorate alone and/or the toxicity chlorite produced by nitrate reductase when chlorite dismutase is absent. In the genome of *H. mediterranei,* a putative gene-encoded chlorite dismutase is present, but no activity has been detected in whole cells or protein extracts. The hyperthermophilic archaeon *Archaeoglobus fulgidus* shows (per)chlorate reduction using a molybdo-enzymes belonging to the type II of DMSO reductases, however chlorite is not enzymatically split into chloride and oxygen [70]. The authors proposed that chlorite is eliminated by an interplay of abiotic and biotic redox reactions involving sulfur compounds and these mechanisms would prevent accumulation of perchlorate in early terrestrial environments. No similar mechanism has been explored yet in haloarchaea.

## 6. DMSO Reductases: Bioremediation with Extremophiles

Bioremediation is the use of microbes for the removal of contaminants of interest or their conversion into less harmful forms. The microbial processes involved in bioremediation are usually related to respiration processes or adaptation strategies, often a component of carbon cycling or metal redox cycling [103]. This section is focused on bioremediation processes that implies the action of DMSO reductases in extreme environments.

### 6.1. (Per)chlorates and Nitrates

The oxyanions perchlorate (ClO_4_^−^) and chlorate (ClO_3_^−^) (collectively, (per)chlorates) are highly soluble strong oxidants with important implications in human health due to their high toxicity [50]. They are deposited in the environment through both anthropogenic and natural processes [104] perchlorate is mainly recognized as a groundwater pollutant derived from activities as pyrotechnics and oil industry [100], contaminating drinking water and food [105]; anthropogenic chlorate is an active component of herbicides among other compounds [104].

To date, the main application for (per)chlorates bioremediation has been related to their anaerobic respiration or dissimilation by microorganisms through (per)chlorate reductases in contaminated waste streams and groundwaters [100]. Due to the importance of (per)chlorates as toxic for human beings, the main research has been focused on drinking water contamination, so most strains of (per)chlorate reducers have been obtained from freshwater, mesophilic, and neutral pH environments [60]. However, one of the most attractive treatments to remove perchlorate from water is the use of ion-exchange techniques, which concentrate this oxyanion in brines [105]. Biological treatment can subsequently remove the perchlorate but using salt-adapted microorganisms. They can be used as a single treatment process in saline wastes or can be applied in combination with ion exchange methods [104,106].

Therefore, in the last years, some halophiles have been described as good per(chlorates) reducers: *Haloferax mediterranei*, *Haloferax denitrificans*, *Haloferax gibbonsii*, *Haloarcula marismortui,* and *Haloarcula vallismortis* [107]. All strains grew in aerobic conditions with perchlorate up to 0.4 M. *H. mediterranei* showed the best phenotypic parameters in these conditions: Its doubling time was less than 4 h and it was the only strain able to growth with 0.6 M perchlorate (the highest concentration tested).

Likewise, many dissimilatory perchlorate-reducing bacteria and archaea can also respire NO_3_^−^ producing N_2_ as a final product [100]. This feature increases the possibilities of using halophilic organisms that can reduce, not only (per)chlorates, but also nitrates. In fact, ion exchange technologies concentrate perchlorate and other ions as nitrates [104]. Nitrate is also present in environments where (per)chlorate is faced as contaminants and its concentrations are increasing annually in such systems due to the use of fertilizers in agriculture [50,108].

The interaction between nitrate and (per)chlorate reductions in bioremediation is complex: On the one hand, some reports state that nitrate inhibits perchlorate reduction completely [109], while others conclude that there is no inhibition when nitrate is present [110] in some cases, they lose the denitrifying ability after cultivation on ClO_3_^−^, while in others, they can recover it after repeat aerobic subculturing in the presence of nitrate [100].

One of the best extremophiles studied in terms of its ability to reduce (per)chlorates and nitrates in anaerobic conditions is *H. mediterranei*. It can reduce perchlorate, chlorate, nitrate as well as bromate through the same enzyme: respiratory nitrate reductase [50]. Moreover, this enzyme permits the reduction of nitrate to dinitrogen with low and transient accumulations of the main toxic intermediates of denitrification, NO and N_2_O [108].

### 6.2. Arsenates and Sulphates

Arsenic (As) is one of the most abundant and ubiquitous toxic heavy metals, and it occurs predominantly in the form of the inorganic oxyanions arsenate (H3AsO4) [As(V)] and arsenite (H3AsO3) [As(III)] [111]. Several groundwaters, sediments, and minerals are enriched in As, which can contaminate fluvial waters, affecting the quality of water resources thus limiting their use [112]. One of the most widely used pathways for the bioremediation of arsenic is the reduction of arsenate to arsenite through arsenate reductase. In these cases, As(V) acts as an electron acceptor and its reduction, coupled with the oxidation of inorganic and organic substrates, leads to generation of energy [113]. Although As(III) is 100 times more toxic than As(V) [114], it is more mobile and it can be removed by precipitation or complexation with sulphide [115]. Extremophiles members of Bacteria and Archaea Domains can reduce arsenate to arsenite as a bioenergetic substrate: examples of this metabolic capacity are some thermophile archaea like *Pyrobaculum aersenaticum* and *Pyrobaculum aerophilum* [116] and bacteria like *Thermus sediminis* [117]. More recently, 18 haloarchaea strains belonging to the Halorubrum genus able to reduce arsenate have been discovered [73].

As mentioned before, sulfate-reducing bacteria/archaea (SRB) are combined with arsenate reducers in bioremediation process. SRB use the sulphate as electron acceptor to produce sulfide [118], which react with As to form a complex with low solubility that precipitates arsenic [115]. Microorganisms that can couple the reduction of arsenate and sulfate are the best candidates for their use in bioremediation processes. The main problem is that certain levels of As decline sulfate removal rates [119]. In this sense, prokaryotic acidophiles have emerged as an interesting group for bioremediation of waters contaminated with sulfates and arsenates with the advantage of their low pH resistance.

Serrano and co-workers demonstrated that a consortium of sulfate-reducing bacteria from Azufre River (Chile) could reduce more than 60% of initial sulfate as well as near 80% of initial As starting from an initial pH of 3.5 [112]. Thus, bioremediation systems that are based on acid/metal-tolerant sulfate-reducing bacteria could be used as new tool for the treatment of these waters.

## 7. Conclusions

DMSO reductases are quite versatile enzymes catalyzing key reactions belonging to several biogeochemical cycles. Although several aspects, like the biosynthesis of the MoCo cofactor, have been extensively described so far, other subjects like the basis of molecular biology related to DMSO operons, phylogeny, and evolution or potential applications of these enzymes at industrial processes remain undescribed. The lack of knowledge on these enzymes is particularly critical in the case of extreme microbes. Bearing in mind that most of the extremophilic microorganisms show genuine metabolisms able to deal with toxic compounds like arsenate, (per)chorate, etc., it seems a contradiction that this type of enzyme has not been better explored from extremophiles to date. Within this context, new questions arise: (i) How relevant are the reactions catalyzed by DMSOs in anoxic environments containing significant concentrations of toxic compounds like arsenate? (ii) How the active sites of DMSOs and MoCo cofactor discriminate between the substrates? (iii) Which are the main amino acidic residues making possible dual activities in the case of NarGHs dealing with nitrate, (per)chlorate, bromate, selenate? More research is required in this field of knowledge in the future, and the potential applications of these enzymes on a large scale would be of high soundness.

## Figures and Tables

**Table 1:** Families of enzymes containing molybdenum or tungsten. Molecules drawn with BioVIA Draw 2019 [8].

Family	Cofactor Structure	Ligands for Coordination	Example of Enzymes
Sulfite oxidase family	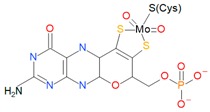	Cysteine	Sulfite oxidases Eukaryotic assimilatory nitrate reductases Sulfite dehydrogenase
Xanthine oxidase family	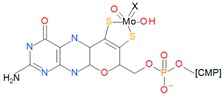	X: Sulfur, Selenium, Oxygen and S-Cu-S(Cys)	Xanthine oxidases Aldehyde oxidases 4-hydroxybenzoyl-CoA reductases Nicotine dehydrogenase
DMSO reductase family	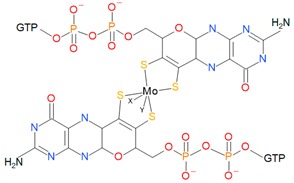	X: Sulfur, Selenium, Oxygen, Y: Aspartate, Serine, Cysteine, and Selenocysteine	DMSO reductases Arsenate reductases Respiratory nitrate reductases Assimilatory nitrate reductases (Per)Chlorate reductases Polysulfide reductases
Tungstoenzymes family	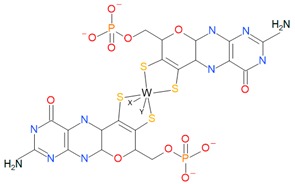	X: Sulfur, Selenium, Oxygen, Y: Aspartate, Serine, Cysteine, and Selenocysteine	Aldehyde oxidoreductases Ferredoxin oxidoreductases Formate dehydrogenses Glyceraldehyde-3-phosphate oxidorreductase

## Abbreviations

FeMoCo	iron-molybdenum Cofactor
MoCo	molybdenum pterin-based Cofactor
cPMP	cyclic pyranopterin monophosphate
5′-GTP	guanosine triphosphate
MPT	molybdopterin / metal-Binding Pterin
FeVCo	iron-vanadium cofactor
WCo	tungsten cofactor
DMSO	dimethyl sulfoxide
mARC	mitochondrial amidoxime reducing components
Bis-MGD	bis-molybdopterin-guanine dinucleotide
3′,8-cH2GTP	3′,8-cyclo-7,8-dihydro-guanosine 5′-triphosphate
Bis-Mo-MPT	bis-mo-molybdopterin
TMAO	trimethylamine N-oxide
NapA	periplasmic nitrate reductase
NarG	respiratory nitrate reductase
Nas	assimilatory nitrate reductase
PsrA	polysulfide reductases
ArrA	respiratory arsenate reductases
Fdh-N	formate dehydrogenases N
PcrA	perchlorate reductases
SerA	respiratory selenate reductases
ClrA	chlorate reductases
DmsA	DMSO reductases
TorA	TMAO reductases
